# 4-Meth­oxy­anilinium hexa­fluoro­phosphate monohydrate

**DOI:** 10.1107/S1600536810022348

**Published:** 2010-06-23

**Authors:** Yong-le Yang, Xue-qun Fu

**Affiliations:** a Ordered Matter Science Research Center, Southeast University, Nanjing 210096, People’s Republic of China

## Abstract

In the structure of the title compound, C_7_H_10_NO^+^·PF_6_^−^·H_2_O, the protonated 4-meth­oxy­anilinium cations and hexa­fluoro­phosphate anions are bridged by the water mol­ecule *via* N—H⋯O and O—H⋯F hydrogen bonds. The resulting zigzag chains extend along the *c* axis. In addition, C—H⋯π inter­actions are observed in the crystal packing.

## Related literature

The title compound was studied as part of our search for ferroelectric compounds, which usually have a phase transition. For background to phase-transition materials, see: Li *et al.* (2008 ▶); Zhang *et al.* (2009 ▶); Fu (2009 ▶). 
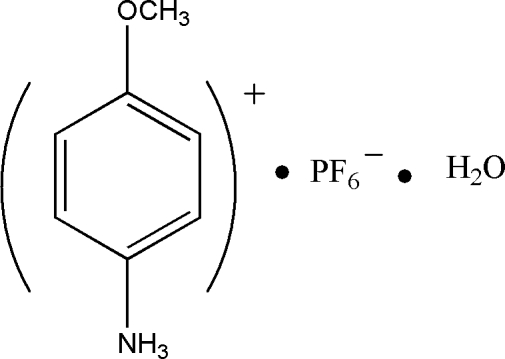


## Experimental

### 

#### Crystal data

C_7_H_10_NO^+^·PF_6_^−^·H_2_O
*M* *_r_* = 287.15
Monoclinic, 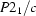

*a* = 15.152 (3) Å
*b* = 5.079 (1) Å
*c* = 14.758 (3) Å
β = 94.26 (3)°
*V* = 1132.6 (4) Å^3^
*Z* = 4
Mo *K*α radiation
μ = 0.32 mm^−1^
*T* = 298 K
0.20 × 0.20 × 0.20 mm

#### Data collection

Rigaku SCXmini diffractometer
Absorption correction: multi-scan (*CrystalClear*; Rigaku, 2005 ▶) *T*_min_ = 0.939, *T*_max_ = 0.939
11166 measured reflections
2602 independent reflections
2083 reflections with *I* > 2σ(*I*)
*R*_int_ = 0.052

#### Refinement

*R*[*F* ^2^ > 2σ(*F* ^2^)] = 0.053
*wR*(*F* ^2^) = 0.145
*S* = 1.06
2602 reflections
199 parameters
3 restraints
H atoms treated by a mixture of independent and constrained refinement
Δρ_max_ = 0.45 e Å^−3^
Δρ_min_ = −0.43 e Å^−3^

### 

Data collection: *CrystalClear* (Rigaku, 2005 ▶); cell refinement: *CrystalClear*; data reduction: *CrystalClear*; program(s) used to solve structure: *SHELXS97* (Sheldrick, 2008 ▶); program(s) used to refine structure: *SHELXL97* (Sheldrick, 2008 ▶); molecular graphics: *SHELXTL* (Sheldrick, 2008 ▶); software used to prepare material for publication: *PRPKAPPA* (Ferguson, 1999 ▶).

## Supplementary Material

Crystal structure: contains datablocks I, global. DOI: 10.1107/S1600536810022348/im2206sup1.cif

Structure factors: contains datablocks I. DOI: 10.1107/S1600536810022348/im2206Isup2.hkl

Additional supplementary materials:  crystallographic information; 3D view; checkCIF report

## Figures and Tables

**Table 1:** Hydrogen-bond geometry (Å, °)

*D*—H⋯*A*	*D*—H	H⋯*A*	*D*⋯*A*	*D*—H⋯*A*
N1—H1*C*⋯O1*W*	0.84 (3)	2.06 (3)	2.896 (3)	172 (3)
N1—H1*A*⋯O1*W*^i^	0.92 (3)	2.00 (3)	2.917 (3)	172 (3)
N1—H1*B*⋯F3^ii^	0.87 (3)	2.32 (3)	3.056 (5)	142 (2)
N1—H1*B*⋯F1^iii^	0.87 (3)	2.49 (3)	3.049 (5)	123 (2)
O1*W*—H1*WB*⋯F6^iv^	0.85 (2)	2.21 (4)	2.91 (2)	139 (3)
O1*W*—H1*WB*⋯F4^v^	0.85 (2)	2.57 (4)	3.04 (3)	116 (3)
C7—H7*B*⋯*Cg*1^vi^	0.96	3.18	4.013 (5)	146

Symmetry codes: (i) 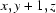; (ii) 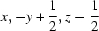; (iii) 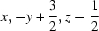; (iv) 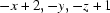; (v) 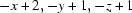; (vi) 
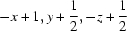
.

**Fig. 1.**
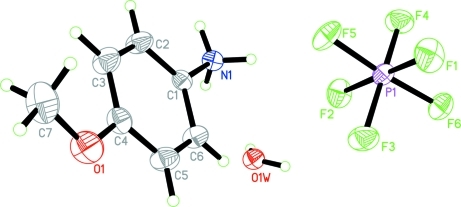
Molecular structure of the title compound, with the atomic numbering scheme. Displacement ellipsoids are drawn at the 30% probability level.

**Fig. 2.**
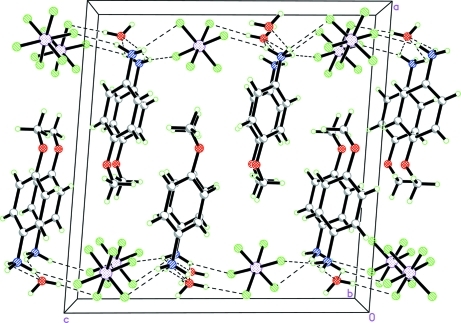
View of the packing of the title compound, stacking along the b axis. Dashed lines indicate hydrogen bonds.

## supplementary crystallographic information

### Comment

As a continuation of our study of dielectric-ferroelectric materials, including organic ligands (Li *et al.*, 2008), metal-organic coordination compounds (Zhang *et al.*, 2009) and organic-inorganic hybrid materials, we studied the dielectric properties of the title compound. Unfortunately, there was no distinct anomaly observed from 93 K to 350 K, suggesting that this compound should be not a real ferroelectric material or there may be no distinct phase transition occurred within the measured temperature range. The crystal structure of 4-methoxyanilinium bromide is known (Fu, 2009). In this article, the crystal structure of the title compound is presented.

The asymmetric unit of the title compound consists of an almost planar 4-methoxyanilinium cation with a mean deviation from the plan of 0.0512 Å, a disordered hexafluorophosphate anion and a water molecule (Fig.1). N—H···F, N—H···O and O—H···F hydrogen bonds link the cations, anions and water molecules to chains along *c* axis (Fig.2). The C—H···π interactions with a C7···*Cg*1 distance of 4.013 (5) Å are also observed in the crystal packing.

### Experimental

1.23 g (10 mmol) 4-Methoxyaniline was dissolved in 10 ml e thanol, to which hexafluorophosphoric acid in aqueous solution (70% *w*/*w*) was then added under stirring until the pH of the solution was *ca* 6. Ethanol was added until all suspended substrates disappeared. Single crystals of the title compound were prepared by slow evaporation of the acidic solution at room temperature of the acidic solution after 3 days giving a yield of 85%.

### Refinement

Positional parameters of all the H atoms bonded to C atoms were calculated geometrically and were allowed to ride on the C atoms to which they are bonded, with *U*_iso_(H) = 1.2*U*_eq_(C) and *U*_iso_(H) = 1.5*U*_eq_(C) for the methyl group. H atoms bonded to N and O atoms were found in the difference Fourier maps and were refined using restraints for O—H and N—H bond distances (0.85–0.86 Å) and angles at the corresponding O and N atoms. Thermal parameters of these hydrogen atoms were refined freely.

### Crystal data

**Table:**

C_7_H_10_NO^+^·PF_6_^−^·H_2_O	*F*(000) = 584
*M**_r_* = 287.15	*D*_x_ = 1.684 Mg m^−^^3^
Monoclinic, *P*2_1_/*c*	Mo *K*α radiation, λ = 0.71073 Å
Hall symbol: -P 2ybc	Cell parameters from 5000 reflections
*a* = 15.152 (3) Å	θ = 3.1–27.6°
*b* = 5.079 (1) Å	µ = 0.32 mm^−^^1^
*c* = 14.758 (3) Å	*T* = 298 K
β = 94.26 (3)°	Prism, colourless
*V* = 1132.6 (4) Å^3^	0.20 × 0.20 × 0.20 mm
*Z* = 4	

### Data collection

**Table:**

Rigaku SCXmini diffractometer	2602 independent reflections
Radiation source: fine-focus sealed tube	2083 reflections with *I* > 2σ(*I*)
graphite	*R*_int_ = 0.052
Detector resolution: 13.6612 pixels mm^-1^	θ_max_ = 27.5°, θ_min_ = 3.2°
ω scans	*h* = −19→19
Absorption correction: multi-scan (*CrystalClear*; Rigaku, 2005)	*k* = −6→6
*T*_min_ = 0.939, *T*_max_ = 0.939	*l* = −19→18
11166 measured reflections	

### Refinement

**Table:**

Refinement on *F*^2^	Primary atom site location: structure-invariant direct methods
Least-squares matrix: full	Secondary atom site location: difference Fourier map
*R*[*F*^2^ > 2σ(*F*^2^)] = 0.053	Hydrogen site location: inferred from neighbouring sites
*wR*(*F*^2^) = 0.145	H atoms treated by a mixture of independent and constrained refinement
*S* = 1.06	*w* = 1/[σ^2^(*F*_o_^2^) + (0.0694*P*)^2^ + 0.5932*P*] where *P* = (*F*_o_^2^ + 2*F*_c_^2^)/3
2602 reflections	(Δ/σ)_max_ < 0.001
199 parameters	Δρ_max_ = 0.45 e Å^−^^3^
3 restraints	Δρ_min_ = −0.43 e Å^−^^3^

### Special details

**Table:**

Geometry. All e.s.d.'s (except the e.s.d. in the dihedral angle between two l.s. planes) are estimated using the full covariance matrix. The cell e.s.d.'s are taken into account individually in the estimation of e.s.d.'s in distances, angles and torsion angles; correlations between e.s.d.'s in cell parameters are only used when they are defined by crystal symmetry. An approximate (isotropic) treatment of cell e.s.d.'s is used for estimating e.s.d.'s involving l.s. planes.
Refinement. Refinement of *F*^2^ against ALL reflections. The weighted *R*-factor *wR* and goodness of fit *S* are based on *F*^2^, conventional *R*-factors *R* are based on *F*, with *F* set to zero for negative *F*^2^. The threshold expression of *F*^2^ > σ(*F*^2^) is used only for calculating *R*-factors(gt) *etc*. and is not relevant to the choice of reflections for refinement. *R*-factors based on *F*^2^ are statistically about twice as large as those based on *F*, and *R*- factors based on ALL data will be even larger.

### Fractional atomic coordinates and isotropic or equivalent isotropic displacement parameters (Å^2^)

**Table:**

	*x*	*y*	*z*	*U*_iso_*/*U*_eq_	Occ. (<1)
N1	0.83257 (13)	0.3532 (5)	0.34412 (16)	0.0380 (5)	
H1B	0.8411 (18)	0.369 (5)	0.287 (2)	0.044 (7)*	
H1A	0.8573 (19)	0.507 (6)	0.368 (2)	0.054 (8)*	
H1C	0.858 (2)	0.217 (7)	0.365 (2)	0.059 (9)*	
C1	0.73739 (14)	0.3340 (4)	0.35733 (15)	0.0341 (5)	
C6	0.70834 (17)	0.1498 (5)	0.41677 (18)	0.0464 (6)	
H6A	0.7482	0.0365	0.4479	0.056*	
C4	0.56043 (16)	0.3061 (5)	0.38507 (18)	0.0456 (6)	
C2	0.67959 (18)	0.5003 (5)	0.3115 (2)	0.0523 (7)	
H2A	0.7001	0.6220	0.2709	0.063*	
O1	0.47356 (12)	0.2800 (5)	0.40334 (16)	0.0671 (6)	
C5	0.61892 (17)	0.1359 (6)	0.42951 (19)	0.0520 (7)	
H5A	0.5984	0.0100	0.4686	0.062*	
C3	0.58996 (18)	0.4883 (6)	0.3253 (2)	0.0568 (8)	
H3A	0.5503	0.6025	0.2945	0.068*	
C7	0.4131 (2)	0.4775 (8)	0.3713 (3)	0.0747 (10)	
H7A	0.3550	0.4345	0.3887	0.112*	
H7B	0.4122	0.4878	0.3063	0.112*	
H7C	0.4312	0.6440	0.3973	0.112*	
O1W	0.91135 (11)	−0.1445 (4)	0.40178 (13)	0.0430 (4)	
H1WA	0.925 (2)	−0.165 (7)	0.4589 (12)	0.069 (10)*	
H1WB	0.9587 (16)	−0.128 (8)	0.3745 (18)	0.082 (12)*	
P1	0.87333 (4)	0.53074 (12)	0.62599 (4)	0.0359 (2)	
F5	0.8065 (12)	0.680 (4)	0.5640 (14)	0.067 (5)	0.582 (6)
F3	0.7987 (3)	0.3110 (9)	0.6454 (3)	0.0524 (4)	0.582 (6)
F1	0.8401 (3)	0.7005 (8)	0.7068 (3)	0.0524 (4)	0.582 (6)
F4	0.9546 (19)	0.699 (6)	0.5972 (19)	0.069 (5)	0.582 (6)
F6	0.9439 (11)	0.379 (3)	0.6897 (13)	0.067 (4)	0.582 (6)
F2	0.9045 (3)	0.3475 (11)	0.5434 (3)	0.0524 (4)	0.582 (6)
F3'	0.7951 (4)	0.3803 (12)	0.6680 (4)	0.0524 (4)	0.418 (6)
F5'	0.8045 (13)	0.667 (4)	0.5461 (19)	0.046 (2)	0.418 (6)
F1'	0.8666 (4)	0.7675 (12)	0.6988 (4)	0.0524 (4)	0.418 (6)
F6'	0.9394 (13)	0.388 (4)	0.7046 (18)	0.047 (2)	0.418 (6)
F4'	0.945 (2)	0.740 (8)	0.598 (2)	0.065 (6)	0.418 (6)
F2'	0.8876 (4)	0.3068 (15)	0.5555 (5)	0.0524 (4)	0.418 (6)

### Atomic displacement parameters (Å^2^)

**Table:**

	*U*^11^	*U*^22^	*U*^33^	*U*^12^	*U*^13^	*U*^23^
N1	0.0359 (11)	0.0378 (11)	0.0412 (12)	−0.0003 (9)	0.0080 (9)	0.0002 (9)
C1	0.0338 (11)	0.0351 (11)	0.0340 (11)	−0.0031 (9)	0.0065 (9)	−0.0053 (9)
C6	0.0441 (13)	0.0461 (14)	0.0498 (14)	0.0043 (11)	0.0088 (11)	0.0103 (11)
C4	0.0356 (12)	0.0542 (15)	0.0479 (14)	−0.0046 (11)	0.0088 (10)	−0.0055 (12)
C2	0.0456 (14)	0.0516 (15)	0.0606 (17)	0.0013 (12)	0.0091 (12)	0.0214 (13)
O1	0.0357 (10)	0.0845 (15)	0.0826 (15)	−0.0045 (10)	0.0152 (9)	0.0085 (12)
C5	0.0486 (15)	0.0540 (16)	0.0552 (16)	−0.0036 (12)	0.0156 (12)	0.0134 (13)
C3	0.0411 (14)	0.0623 (18)	0.0668 (18)	0.0074 (13)	0.0021 (13)	0.0176 (14)
C7	0.0395 (15)	0.100 (3)	0.086 (2)	0.0104 (17)	0.0084 (15)	−0.015 (2)
O1W	0.0348 (9)	0.0483 (10)	0.0466 (10)	−0.0002 (8)	0.0074 (8)	0.0041 (8)
P1	0.0356 (3)	0.0405 (3)	0.0323 (3)	0.0051 (2)	0.0072 (2)	0.0037 (2)
F5	0.067 (4)	0.088 (5)	0.045 (8)	0.016 (3)	−0.004 (3)	0.024 (4)
F3	0.0545 (9)	0.0554 (14)	0.0500 (10)	−0.0045 (8)	0.0216 (9)	−0.0070 (6)
F1	0.0545 (9)	0.0554 (14)	0.0500 (10)	−0.0045 (8)	0.0216 (9)	−0.0070 (6)
F4	0.053 (3)	0.074 (11)	0.079 (5)	−0.014 (4)	0.008 (3)	0.016 (5)
F6	0.059 (4)	0.102 (5)	0.039 (6)	0.035 (4)	0.005 (3)	0.008 (3)
F2	0.0545 (9)	0.0554 (14)	0.0500 (10)	−0.0045 (8)	0.0216 (9)	−0.0070 (6)
F3'	0.0545 (9)	0.0554 (14)	0.0500 (10)	−0.0045 (8)	0.0216 (9)	−0.0070 (6)
F5'	0.043 (4)	0.060 (4)	0.034 (7)	0.016 (3)	−0.003 (3)	0.020 (4)
F1'	0.0545 (9)	0.0554 (14)	0.0500 (10)	−0.0045 (8)	0.0216 (9)	−0.0070 (6)
F6'	0.038 (4)	0.068 (5)	0.037 (7)	0.008 (4)	0.006 (3)	0.017 (4)
F4'	0.070 (13)	0.058 (8)	0.065 (6)	−0.033 (10)	−0.001 (7)	0.003 (5)
F2'	0.0545 (9)	0.0554 (14)	0.0500 (10)	−0.0045 (8)	0.0216 (9)	−0.0070 (6)

### Geometric parameters (Å, °)

**Table:**

N1—C1	1.473 (3)	C7—H7B	0.9600
N1—H1B	0.87 (3)	C7—H7C	0.9600
N1—H1A	0.92 (3)	O1W—H1WA	0.860 (17)
N1—H1C	0.84 (3)	O1W—H1WB	0.853 (17)
C1—C2	1.360 (3)	P1—F5	1.516 (17)
C1—C6	1.377 (3)	P1—F2'	1.567 (8)
C6—C5	1.383 (3)	P1—F6	1.571 (17)
C6—H6A	0.9300	P1—F3'	1.576 (7)
C4—O1	1.370 (3)	P1—F4	1.58 (3)
C4—C5	1.370 (4)	P1—F1	1.584 (4)
C4—C3	1.376 (4)	P1—F4'	1.60 (4)
C2—C3	1.390 (4)	P1—F1'	1.621 (6)
C2—H2A	0.9300	P1—F3	1.629 (5)
O1—C7	1.415 (4)	P1—F2	1.631 (6)
C5—H5A	0.9300	P1—F6'	1.64 (2)
C3—H3A	0.9300	P1—F5'	1.67 (2)
C7—H7A	0.9600		
			
C1—N1—H1B	110.7 (18)	F4—P1—F1	101.9 (11)
C1—N1—H1A	112.3 (18)	F5—P1—F4'	87.0 (17)
H1B—N1—H1A	102 (3)	F2'—P1—F4'	100.4 (14)
C1—N1—H1C	108 (2)	F6—P1—F4'	92.0 (16)
H1B—N1—H1C	110 (3)	F3'—P1—F4'	166.3 (15)
H1A—N1—H1C	113 (3)	F1—P1—F4'	95.4 (14)
C2—C1—C6	121.0 (2)	F5—P1—F1'	87.6 (8)
C2—C1—N1	119.6 (2)	F2'—P1—F1'	175.6 (2)
C6—C1—N1	119.5 (2)	F6—P1—F1'	92.3 (8)
C1—C6—C5	118.9 (2)	F3'—P1—F1'	90.73 (19)
C1—C6—H6A	120.5	F4—P1—F1'	82.4 (11)
C5—C6—H6A	120.5	F4'—P1—F1'	75.9 (14)
O1—C4—C5	116.2 (2)	F5—P1—F3	90.4 (8)
O1—C4—C3	123.7 (3)	F2'—P1—F3	75.58 (18)
C5—C4—C3	120.1 (2)	F6—P1—F3	90.6 (5)
C1—C2—C3	120.0 (2)	F4—P1—F3	168.8 (12)
C1—C2—H2A	120.0	F1—P1—F3	88.68 (14)
C3—C2—H2A	120.0	F4'—P1—F3	175.0 (12)
C4—O1—C7	118.2 (2)	F1'—P1—F3	108.24 (18)
C4—C5—C6	120.5 (2)	F5—P1—F2	93.0 (8)
C4—C5—H5A	119.7	F6—P1—F2	86.9 (8)
C6—C5—H5A	119.7	F3'—P1—F2	106.6 (2)
C4—C3—C2	119.4 (3)	F4—P1—F2	80.3 (11)
C4—C3—H3A	120.3	F1—P1—F2	177.73 (15)
C2—C3—H3A	120.3	F4'—P1—F2	86.8 (14)
O1—C7—H7A	109.5	F1'—P1—F2	162.7 (2)
O1—C7—H7B	109.5	F3—P1—F2	89.06 (16)
H7A—C7—H7B	109.5	F5—P1—F6'	172.2 (12)
O1—C7—H7C	109.5	F2'—P1—F6'	92.5 (9)
H7A—C7—H7C	109.5	F3'—P1—F6'	86.9 (6)
H7B—C7—H7C	109.5	F4—P1—F6'	88.9 (13)
H1WA—O1W—H1WB	109 (2)	F1—P1—F6'	85.4 (10)
F5—P1—F2'	94.6 (9)	F4'—P1—F6'	94.9 (16)
F5—P1—F6	179.0 (9)	F1'—P1—F6'	85.6 (9)
F2'—P1—F6	85.5 (8)	F3—P1—F6'	88.3 (5)
F5—P1—F3'	89.5 (8)	F2—P1—F6'	94.7 (10)
F2'—P1—F3'	93.1 (2)	F2'—P1—F5'	86.7 (9)
F6—P1—F3'	91.5 (6)	F6—P1—F5'	171.6 (12)
F5—P1—F4	93.9 (14)	F3'—P1—F5'	91.7 (8)
F2'—P1—F4	93.7 (11)	F4—P1—F5'	92.6 (14)
F6—P1—F4	85.1 (13)	F1—P1—F5'	95.1 (9)
F3'—P1—F4	172.2 (10)	F4'—P1—F5'	86.6 (16)
F5—P1—F1	86.9 (8)	F1'—P1—F5'	95.4 (9)
F2'—P1—F1	164.18 (19)	F3—P1—F5'	90.2 (8)
F6—P1—F1	93.3 (8)	F2—P1—F5'	84.8 (9)
F3'—P1—F1	71.17 (18)	F6'—P1—F5'	178.4 (10)
			
C2—C1—C6—C5	−0.1 (4)	O1—C4—C5—C6	−178.9 (3)
N1—C1—C6—C5	179.2 (2)	C3—C4—C5—C6	1.7 (4)
C6—C1—C2—C3	0.9 (4)	C1—C6—C5—C4	−1.3 (4)
N1—C1—C2—C3	−178.3 (3)	O1—C4—C3—C2	179.8 (3)
C5—C4—O1—C7	169.6 (3)	C5—C4—C3—C2	−0.8 (4)
C3—C4—O1—C7	−11.0 (4)	C1—C2—C3—C4	−0.5 (5)

### Hydrogen-bond geometry (Å, °)

**Table:**

*D*—H···*A*	*D*—H	H···*A*	*D*···*A*	*D*—H···*A*
N1—H1C···O1W	0.84 (3)	2.06 (3)	2.896 (3)	172 (3)
N1—H1A···O1W^i^	0.92 (3)	2.00 (3)	2.917 (3)	172 (3)
N1—H1B···F3^ii^	0.87 (3)	2.32 (3)	3.056 (5)	142 (2)
N1—H1B···F1^iii^	0.87 (3)	2.49 (3)	3.049 (5)	123 (2)
O1W—H1WB···F6^iv^	0.85 (2)	2.21 (4)	2.91 (2)	139 (3)
O1W—H1WB···F4^v^	0.85 (2)	2.57 (4)	3.04 (3)	116 (3)
C7—H7B···Cg1^vi^	0.96	3.18	4.013 (5)	146

Symmetry codes: (i) *x*, *y*+1, *z*; (ii) *x*, −*y*+1/2, *z*−1/2; (iii) *x*, −*y*+3/2, *z*−1/2; (iv) −*x*+2, −*y*, −*z*+1; (v) −*x*+2, −*y*+1, −*z*+1; (vi) −*x*+1, *y*+1/2, −*z*+1/2.
